# Piezoelectric Nanomaterials Activated by Ultrasound: The Pathway from Discovery to Future Clinical Adoption

**DOI:** 10.1021/acsnano.1c03087

**Published:** 2021-07-12

**Authors:** Andrea Cafarelli, Attilio Marino, Lorenzo Vannozzi, Josep Puigmartí-Luis, Salvador Pané, Gianni Ciofani, Leonardo Ricotti

**Affiliations:** † The BioRobotics Institute, Scuola Superiore Sant’Anna, 56127 Pisa, Italy; ‡ Department of Excellence in Robotics & AI, Scuola Superiore Sant’Anna, 56127 Pisa, Italy; § Smart Bio-Interfaces, Istituto Italiano di Tecnologia, 56025 Pontedera, Italy; ∥ Departament de Ciència dels Materials i Química Física, Institut de Química Teòrica i Computacional, 08028 Barcelona, Spain; ⊥ Institució Catalana de Recerca i Estudis Avançats (ICREA), 08010 Barcelona, Spain; # Multi-Scale Robotics Lab (MSRL), Institute of Robotics and Intelligent Systems (IRIS), ETH Zurich, 8092 Zurich, Switzerland

**Keywords:** piezoelectric nanomaterials, ultrasound, electric stimuli, piezoelectric effect, mechanoelectrical transduction, neuromodulation, regenerative medicine, cancer treatment

## Abstract

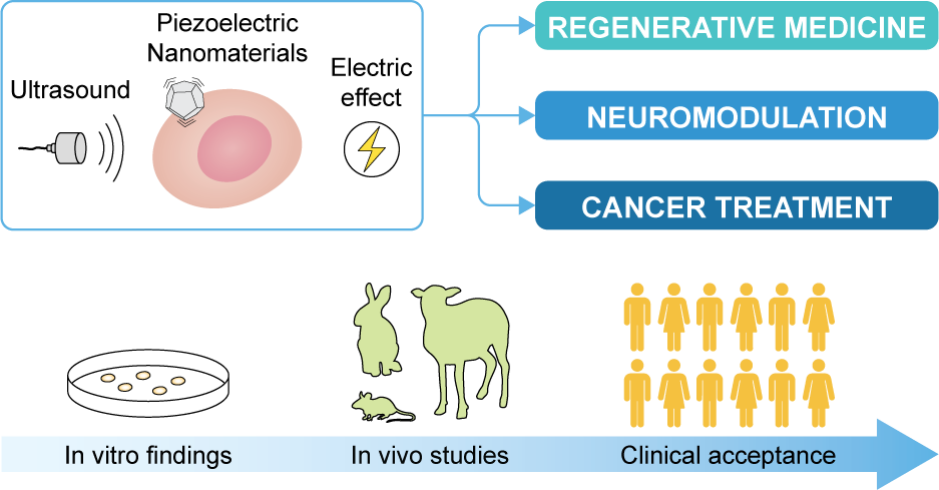

Electrical stimulation has shown great promise in biomedical applications, such as regenerative medicine, neuromodulation, and cancer treatment. Yet, the use of electrical end effectors such as electrodes requires connectors and batteries, which dramatically hamper the translation of electrical stimulation technologies in several scenarios. Piezoelectric nanomaterials can overcome the limitations of current electrical stimulation procedures as they can be wirelessly activated by external energy sources such as ultrasound. Wireless electrical stimulation mediated by piezoelectric nanoarchitectures constitutes an innovative paradigm enabling the induction of electrical cues within the body in a localized, wireless, and minimally invasive fashion. In this review, we highlight the fundamental mechanisms of acoustically mediated piezoelectric stimulation and its applications in the biomedical area. Yet, the adoption of this technology in a clinical practice is in its infancy, as several open issues, such as piezoelectric properties measurement, control of the ultrasound dose *in vitro*, modeling and measurement of the piezo effects, knowledge on the triggered bioeffects, therapy targeting, biocompatibility studies, and control of the ultrasound dose delivered *in vivo*, must be addressed. This article explores the current open challenges in piezoelectric stimulation and proposes strategies that may guide future research efforts in this field toward the translation of this technology to the clinical scene.

## Introduction

Endogenous electric fields play a crucial role in cellular physiology, not only in the generation and propagation of the action potentials in nerves and muscles but also in controlling other cellular functions, such as proliferation, morphology, gene expression, differentiation, and migration.^1^ As a therapeutic tool, therefore, electrical stimulation has exciting potential in different biomedical applications, such as neuromodulation, regenerative medicine, and cancer treatment.

To date, therapies based on electrical stimuli require invasive percutaneous electrodes or transcutaneous devices, which typically lack efficacy and spatial resolution. In this vein, piezoelectric nanoparticles activated by external ultrasound (US) constitute a paradigm enabling the induction of localized electrical stimulation within the body in a wireless fashion. Indeed, electrical stimuli can be conveyed on-demand and with high spatial precision into the target tissue using an external mechanical source, such as a US transducer, and exploiting the piezoelectric properties of nanoparticles exposed to such mechanical stress.

US is widely accepted in medicine for both diagnostic purposes^2^ and therapeutic ones,^3^ in which they are used to induce thermal or mechanical effects in the target area, causing tissue destruction^4^ or tissue modifications/repair.^5^ Piezoelectric nanomaterials also find their application in a wide variety of biomedical fields,^6^ including sensors and actuators^7,8^ and energy-harvesting systems.^9,10^ However, the idea to combine them by remotely activating piezoelectric particles with external US to produce electrical charges *in situ* is a relatively young research area. In this scenario, US waves are exploited to mechanically activate the nanoparticles, thus remotely generating electrical charges within tissues by exploiting the direct piezoelectric effect.^11^

**Figure 1:**
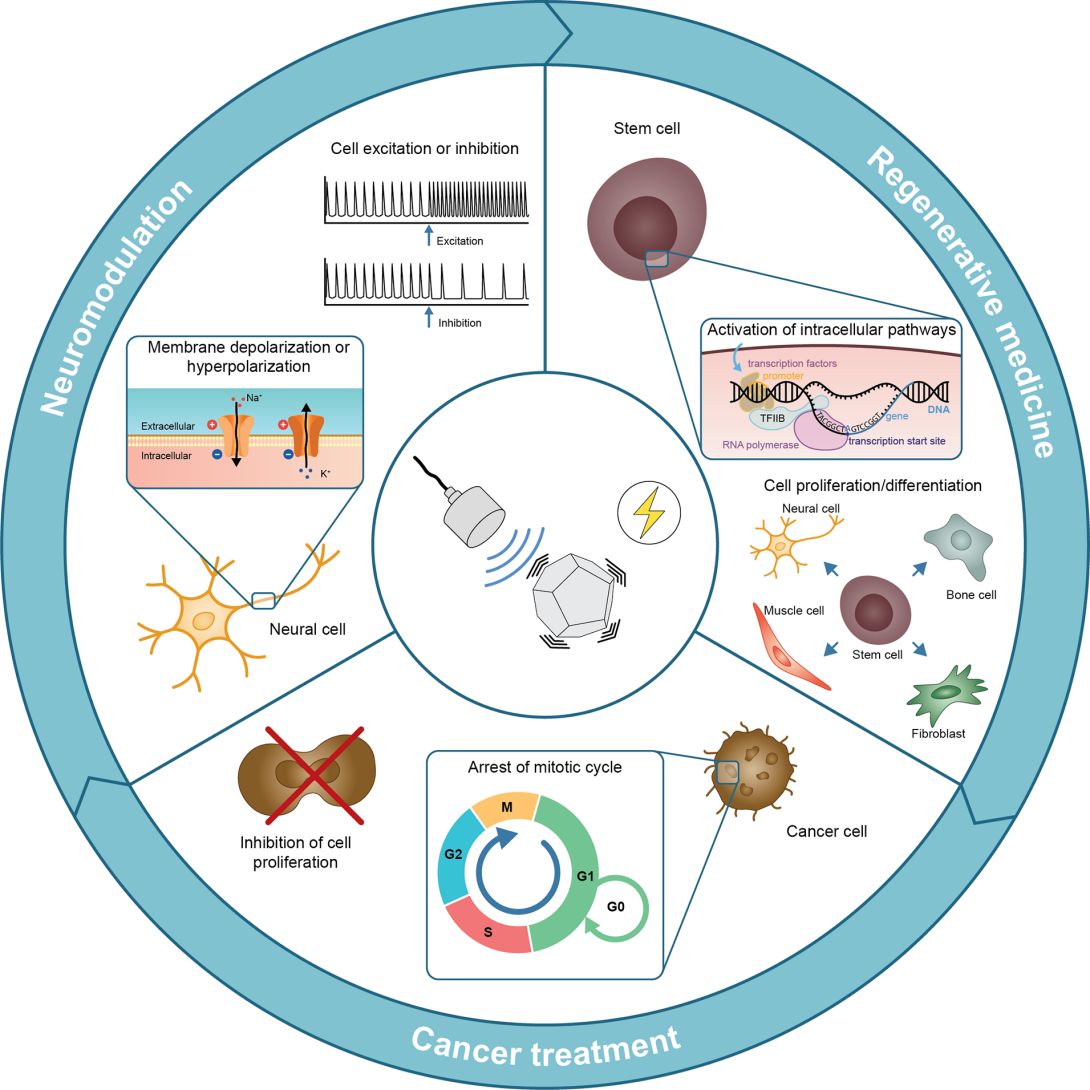
Scheme of the “US-activated piezoelectric nanoparticle stimulation” paradigm and the main research domains in which this paradigm is nowadays explored. US waves can interact with piezoelectric nanoparticles, generating a localized electrical stimulation used for neuromodulation, regeneration, or cancer treatment purposes.

Recent investigations have shown several biological effects triggered by US-stimulated piezoelectric nanoparticles (*i.e.*, neural modulation, proliferation, or inhibition of different cell lines and differentiation of stem cells—see next section), thus continuously increasing the scientific interest in indirect electrical stimulation achieved through piezoelectric nanoparticles that act as nanotransducers at the tissue and cell level. Figure 1 schematically depicts the general concept and the different research domains in which the “US-activated piezoelectric nanoparticle stimulation” paradigm has shown its highest potential.

In this review, we describe the fundamental mechanisms and main applications that have been described in the state-of-the-art, so far, concerning the interaction between piezoelectric nanoparticles and US waves. The future *in vivo* translation of these findings is not trivial: the adoption of this paradigm in clinical practice will be possible only once some open issues will be convincingly addressed by the scientific community. This paper summarizes some of the current open challenges in piezoelectric stimulation for biomedical applications and proposes approaches that may guide future research efforts in this field, enabling the translation of this technology from the bench to the clinics.

## Physics of Piezoelectric Nanomaterials and Ultrasound Waves

### Piezoelectric Nanomaterials

**Figure 2:**
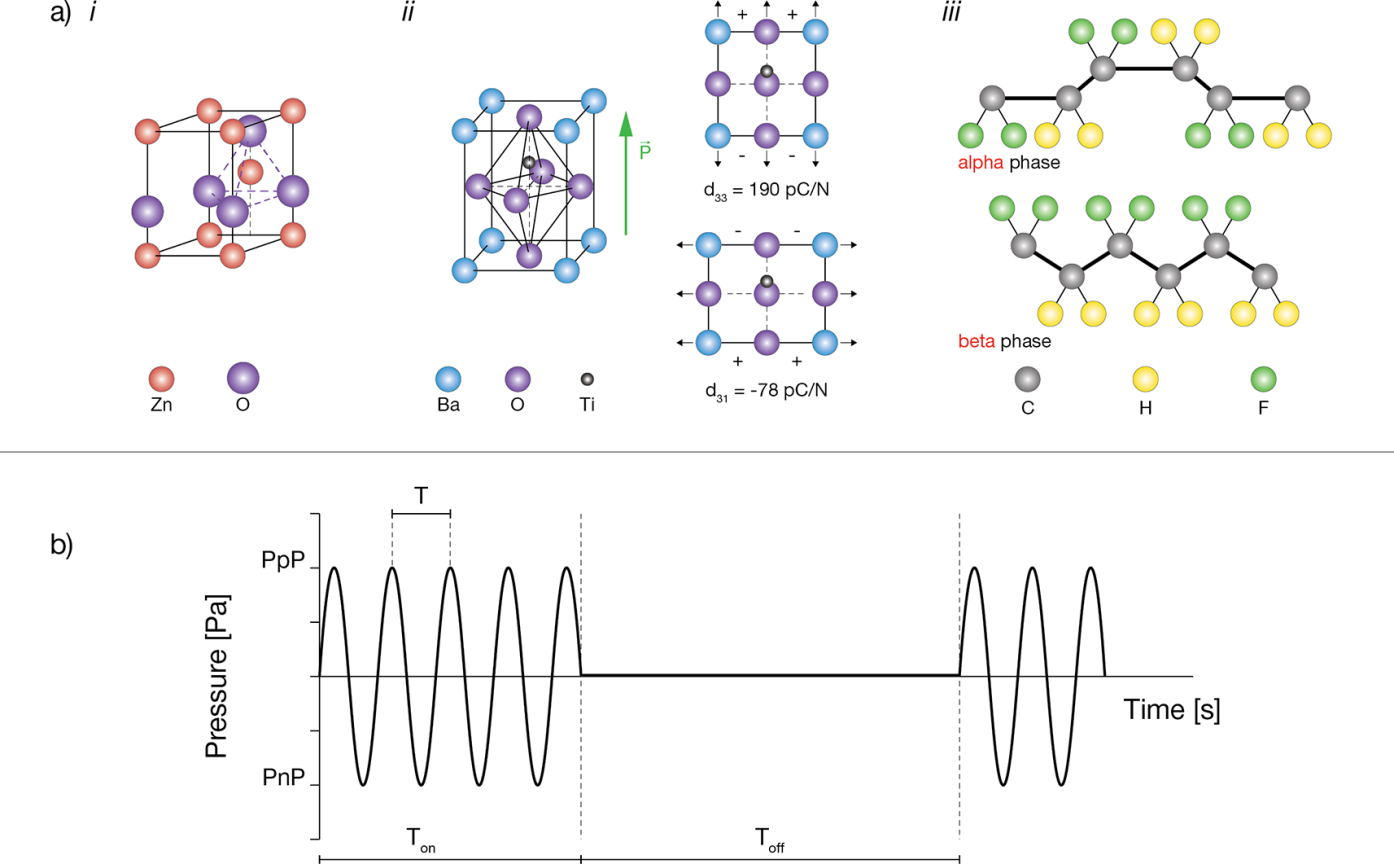
(a) Unit cells of characteristic piezoelectric materials. (i) ZnO: inorganic (nonferroelectric) material with a wurtzite structure. Image adapted with permission from ref (53). Copyright 2009 Elsevier. (ii) BaTiO_3_: inorganic (ferroelectric) ceramic material with a perovskite structure. The piezoelectric coefficients are defined with respect to the applied stress direction. Image adapted with permission from ref (54). Copyright 2002 John Wiley and Sons. (iii) PVDF: synthetic organic polymer exhibiting high piezoelectricity in the β structure. Image adapted with permission from ref (7). Copyright 2019 John Wiley and Sons. (b) Typical features of a pulsed ultrasound wave at a specific frequency (*f*): period (*T* = 1/*f*), pulse period time (*T*_on_), delay time (*T*_off_), peak of positive pressure (*P*_pP_), and peak of negative pressure (*P*_nP_). These parameters also allow calculating the pulse repetition period (*P*_RP_ = *T*_on_ + *T*_off_), duty cycle (DC = *T*_on_/*P*_RP_), and burst rate (BR = 1/*P*_RP_); intensity (*I*) is derived by dividing the square of the pressure (*P*_US_) by the density of the medium (ρ) and the traveling wave speed (*c*).

Piezoelectric materials are a subset of inorganic and organic dielectric compounds characterized by their ability to become electrically polarized when they are mechanically stimulated, and *vice versa*, they strain when they are subject to electric fields. Among the existing 32 crystal classes, 21 are noncentrosymmetric, of which 20 are piezoelectric. Figure 2a shows examples of unit cells of both inorganic and organic piezoelectric materials. In inorganic compounds, the piezoelectricity arises due to a relative displacement of ionic species, while a repositioning of molecular dipoles in organic materials occurs.^7^ Within the piezoelectric family, ferroelectric materials exhibit an in-built spontaneous electrical polarization. Ten crystal classes display ferroelectricity, whereas the other 10 are nonferroelectric piezoelectric. Figure 2a(*i*,*ii*) shows the crystal lattices of two inorganic materials, zinc oxide (ZnO), a nonferroelectric piezoelectric, and barium titanate (BaTiO_3_), a ferroelectric material. The latter exhibits a perovskite structure, in which Ba^2+^ ions occupy the vertices of the cubic cell, O^2–^ is located at the center of the cube forming an octahedron, and T^4+^ is slightly shifted up from the center of the cubic cell with respect to the O^2–^ anions. This asymmetry results in a spontaneous electric polarization. In contrast, ZnO, whose piezoelectric form crystallizes in the wurtzite structure,^12^ does not display an in-built electric polarization unless the lattice is mechanically deformed. Figure 2a(*iii*) also shows an example of a synthetic organic polymer, poly(vinylidene) fluoride (PVDF). This polymer exhibits different crystalline structures; among them, the β structure is the crystalline form with the highest piezoelectric coefficient due to the arrangement of the highly electronegative fluorine atoms on the same side of the carbon chain. To maximize this type of crystallinity, PVDF is usually copolymerized with trifluoroethylene (TrFE) to form P(VDF-TrFE). Several natural materials, such as silk, amino acids, collagen, peptides, *etc.*, also display piezoelectric properties. These materials often mediate the transfer of electricity throughout animal tissues, which plays a crucial role in both developing (during embryogenesis) and healing or adapting (in adult organisms) many tissue types.

The applied stress *T* and the resulting electric polarization *P* in a piezoelectric material are proportionally related as follows:
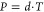
1where *d* corresponds to the piezoelectric coefficient tensor. The piezoelectric coefficients are a function of the material composition and depend on the crystal direction. They are indicated as *d*_*ij*_, where the subscripts *i* and *j* indicate the directions of the generated polarization and the applied stress, respectively. Figure 2a(*ii*) shows two piezoelectric coefficients of BaTiO_3_ corresponding to the polarization induced in a specific direction when the stress is applied in two different directions of the crystal lattice. Table 1 shows different inorganic and organic materials and their corresponding range of piezoelectric coefficients potentially achievable.

**Table 1:** Comparison of Different Piezoelectric Materials in Terms of Type, Structure, and Piezoelectric Coefficients

piezoelectric material	material type	material structure	piezoelectric coefficients
gallium nitride (GaN)	synthetic crystal, nonferroelectric	wurtzite	*d*_33_ = 2–4 [pC/N]
			*d*_31_ = −1.5 to −1.9 [pC/N]^7,13^
aluminum nitride (AlN)	synthetic crystal, nonferroelectric	wurtzite	*d*_33_ = 3–6 [pC/N]
			*d*_31_ = −2 to −2.8 [pC/N]^7,13,14^
lithium niobate (LiNbO_3_)	synthetic crystal, ferroelectric	perovskite	*d*_33_ = 16–41.5 [pC/N]
			*d*_31_ = −1 [pC/N]^7,15^
boron nitride (BN)	synthetic crystal, nonferroelectric	wurtzite	*d*_33_ = 0.3 [pC/N]
			*d*_11_ = 0.5–1.27 [pC/N]^16^
lead zirconate titanate (PZT)	synthetic ceramic, ferroelectric	perovskite	*d*_33_ = 225–590 [pC/N]
			*d*_31_ = −93.5 to −274 [pC/N]^7,17^
zinc oxide (ZnO)	synthetic ceramic, nonferroelectric	wurtzite	*d*_33_ = 3–20 [pC/N]
			*d*_31_ = −5 [pC/N]^7,18,19^
barium titanate (BaTiO_3_)	synthetic ceramic, ferroelectric	perovskite	*d*_33_ = 90–788 [pC/N]
			*d*_31_ = −33.4 to −78 [pC/N]^7,20,21^
potassium–sodium niobate (KNN)	synthetic lead-free, ferroelectric	perovskite	*d*_33_ = 93–700 [pC/N]^22−24^
polyvinylidene fluoride (PVDF)	synthetic polymer, ferroelectric	polymeric (semicrystalline)	*d*_33_ = −20 to −33 [pC/N]
			*d*_31_ = 23 [pC/N]^7,25^
polyvinylidene fluoride-trifluoroethylene (PVDF-TrFE)	synthetic polymer, ferroelectric	polymeric (semicrystalline)	*d*_33_ = 21.5–74 [pC/N]^26,27^
polyhydroxybutyrate (PHB)	synthetic polymer, ferroelectric	polymeric (anisotropic)	*d*_33_ = 2.1–2.5 [pC/N]
			*d*_14_ = 1–2 [pC/N]^22,28^
nylon-11	synthetic polymer, ferroelectric	polymeric (semicrystalline)	*d*_33_ = 3.8–4 [pC/N]
			*d*_31_ = 14 [pC/N]^29,30^
poly-l-lactic acid (PLLA)	synthetic polymer, nonferroelectric	polymeric (semicrystalline)	*d*_33_ = 3.1 [pC/N]
			*d*_31_ = 1.58 [pC/N]
			*d*_14_ = 6–12 [pC/N]^7,29,31^
β-glycine	natural material, nonferroelectric	polymeric (crystalline)	*d*_16_ = 195 [pm/V]^7^
collagen	natural material, nonferroelectric	polymeric (semicrystalline)	*d*_14_ = 0.1 [pm/V]
			*d*_15_ = ∼2 [pC/N]^7,16^
silk	natural material, nonferroelectric	polymeric (semicrystalline)	*d*_14_ = −1.5 [pC/N]^7^
peptide microtubes	natural material, nonferroelectric	self-assembled diphenylalanine dipeptides	*d*_15_ = 60 [pm/V]^7^

Note that miniaturizing piezoelectric materials down to the nanoscale has non-negligible consequences. Due to several factors, such as concentration and elimination of crystal surface defects^32^ or crystal lattice contraction or expansion, nanoscale piezoelectric structures can undergo an increase or a decrease in the piezoelectric figures of merit, with respect to their macroscale counterparts. For example, it has been reported that nonferroelectric piezoelectric nanostructures, such as ZnO nanobelts, exhibit piezoelectric coefficients that are larger than those of bulk ZnO.^33^ In ferroelectric piezoelectric materials, gradual elimination of the spontaneous polarization can occur when shrinking the material size due to crystal lattice contraction.^34^ Additionally, thermal vibrations can cause a permanent switching of the electric dipoles in nanostructures, leading to a zero spontaneous polarization. The mechanical properties such as elastic modulus and toughness of piezoelectric nanomaterials can also be considerably enhanced at the nanoscale.^32^

Two main approaches have been used to structure piezoelectric materials at the nanoscale, namely, top-down and bottom-up methods. While top-down methods such as electron-beam-assisted approaches can guarantee reasonable control on the structure size and position,^35^ they frequently lead to a high concentration of defects, which reduce the piezoelectric coefficient of the final structures. Differently, bottom-up approaches can lead to piezoelectric structures with a smaller size and reduced defect densities. For example, hydrothermal chemical synthesis has been used to yield single crystals of piezoelectric materials with nanorod, nanowire, and/or nanoparticle shapes.^36^ Other bottom-up methods such as sol–gel synthesis have also proven efficient to yield piezoelectric nanoparticles.^37^ All these methods have been used indistinctively to nanostructure piezoelectric materials with a perovskite structure, such as lead zirconate titanate (PZT) and BaTiO_3_, as well as piezoelectric materials with a wurtzite structure, *e.g.*, ZnO.

Recently, the refinement of fabrication techniques allowed nanomaterial piezoelectric coefficients to be to enhanced by poling the materials. This enabled controlling phase and crystallographic orientation, thus facilitating the polarization rotation between different states under an external field. For example, the KNN particle fabrication procedure has been improved to obtain a piezoelectric coefficient up to 700 pC/N by optimizing its anisotropic feature and the domain configuration in textured ceramics, facilitating the design of high-performance piezoelectric devices.^23^

Biocompatibility is an important feature to be considered for the piezoelectric materials listed in Table 1. Since 2014, the Restriction of Hazardous Substances (RoHS) Directive (2011/65/EU, also known as RoHS II) has been applied to medical devices. This regulation standardizes the use of hazardous materials with the objective of limiting the presence of toxic elements (*e.g.*, Pb in PZT). As a consequence, despite the interesting piezoelectric properties of PZT, its use as implantable material is still a matter of debate.^7^ Some attempts have been recently made to make PZT more biocompatible, *e.g.*, by treating its surface with titanium.^38^ In general, inorganic and perovskite/wurtzite piezoelectric materials (*e.g.*, AlN, LiNbO_3_, ZnO) display biocompatibility characteristics or can be turned into biocompatible materials through specific processing, encapsulation, or coating.^39^ On the other hand, organic polymers exhibit biocompatibility features. Anyhow, for a safe application, there is always the need to evaluate the material’s biocompatibility, as it also depends on its shape, size, and external environment. Further considerations on the biocompatibility characteristics of piezoelectric nanomaterials are reported in the Outlook section.

The functionalization of nanoscale piezoelectric materials with biotargeting groups may facilitate the translation of piezoelectric materials to a wealth of *in vivo* applications. So far, both covalent attachment and noncovalent polymer wrapping have been pursued. Exploiting the hydroxyl groups existing at the surface of piezoelectric metal oxides, covalent bonding can be achieved employing a coupling agent-based reaction. Silanes, phosphates, carboxylates, and titanates are coupling agents commonly used to modify oxide surfaces. For example, in this context, 3-glycidoxypropyltrimethoxysilane (GPTMS) and γ-aminopropyl trimethoxysilane (γ-APS) have been used to introduce epoxy or amino groups on the surface of barium titanate.^40,41^ Reagents such as *n-*hexylphosphonic acid (HPA) or pentafluorobenzylphosphonic acid (PFPA) have been also used to effectively modify the surface of barium titanate nanoparticles (BTNPs) with acid groups.^42,43^ These derivatizations with coupling agents that exhibit additional reactive moieties (*i.e.*, amino (−NH_2_) or acid (−COOH) functional groups) enable further derivatization with biotargeting groups such as oligonucleotides, proteins, and/or enzymes. Noncovalent polymer wrapping has also been pursued on piezoelectric nanoparticles, using poly-l-lysine, glycol chitosan, gum Arabic, and other biofriendly molecules, thus facilitating nanoparticle internalization within the target cells, a factor that is crucial to trigger the desired bioeffect, as highlighted in the next sections.

### Ultrasound Waves

US is a mechanical wave (Figure 2b), with frequencies higher than 20 kHz (that is the upper limit of the human hearing range). Unlike electromagnetic waves, US ones cannot travel in the vacuum: they necessarily need a medium to propagate.

A general analytical approach for describing US traveling waves in a compressing medium can be derived by combining momentum, mass, and energy conservation equations.^44^ Under the assumptions of a quiescent and isotropic medium, the single second-order wave equation in a single acoustic variable (*i.e.*, the acoustic pressure, *P*_US_) can be therefore derived as follows:
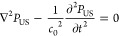
2where *c*_0_ represents the speed of the wave in the medium.

Besides the well-known use of US for diagnostic purposes, therapeutic US recently emerged as a tool to induce beneficial bioeffects within the body in a wireless and intrinsically safe manner. When a US wave interacts with biological tissues, there are two main physical effects: thermal and mechanical ones. They depend on the nature of the tissue (attenuation coefficient, percentage of gas, *etc.*) and the US parameters (intensity, therapy duration, duty cycle, *etc.*). Thermal effects are associated with the deposition in the tissue of part of the energy carried by the US wave. The absorbed ultrasound energy (*i.e.*, the rate of heat deposition per unit volume, *Q̇*) is given by the following equation:
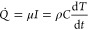
3and it depends on the absorption coefficient (μ) and the acoustic intensity (*I*). If no conduction, convection, or radiation transfer energy are considered, the rate of temperature rise (d*T*/d*t*) can be easily determined by knowing the density of the medium (ρ) and the heat capacity (*C*) of the tissue.

The thermal index (*ThI*) provides a simplified way to estimate the temperature rise in tissue during US exposure, and it is defined as the ratio between the transmitted power (*W*_p_) at the depth of interest and the power needed to raise the tissue temperature by 1 °C (*W*_deg_).
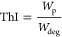
4

Even though it represents a rough estimation of induced thermal effects, *ThI* is currently widely adopted in ultrasound medical devices as proxies of possible thermal risks. Being associated with the total energy deposited in tissues, in order to limit the *ThI*, particular attention should be paid to the employed US parameters, such as intensity, duration, and duty cycle.

Among the mechanical effects induced by US on tissues, the most common ones are related to radiation force, acoustic streaming, and acoustic cavitation. The latter involves the formation, oscillation, and possible collapse of gas bubbles within the tissue. The mechanical index (*MI*), defined as the ratio of the peak negative pressure (*P*_nP_) to the square root of the frequency (*f*), indicates the probability of incepting mechanically induced bioeffects.
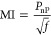
5

If correctly tuned, therapeutic US could therefore produce different thermal and/or mechanical effects within tissues, thus triggering specific desired biological effects that can be exploited for a plethora of different clinical indications. US at high intensities is used for destructive (*i.e.*, cell-killing) applications such as cancer treatment.^45^ Tissue necrosis can occur due to high thermal effects (*i.e.*, *T* > 56 °C and prolonged exposures as described in Foley *et al.*)^46^ or lethal mechanical effects at high *MI*, like inertial cavitation ones, which can be exploited to produce irreversible mechanical damages in the target tissue, such as in focused US histotripsy therapies.^47^ Alternatively, at lower pressure levels, US is exploited for nondestructive (*i.e.*, cell-modifying) applications in physiotherapy,^48^ regenerative medicine,^49^ and targeted drug delivery.^50^ In this case, the low total amount of acoustic energy deposited in tissues results in a low thermal rise (typical *T* lower than 43 °C, like in the case of US-induced mild hyperthermia)^51^ or nonlethal mechanical effects at low *MI*, such as stable cavitation ones.^52^ Low intensity pulsed ultrasound (LIPUS) is gaining interest as a nonthermal and noninvasive strategy to induce beneficial effects in tissues, *e.g.*, increasing proliferation and differentiation.^5^ From an engineering viewpoint, an additional possible classification can also be made by distinguishing applications in which US are used alone (direct effect on tissues) or in combination with US-responsive agents (mediated effects on tissues) like in the case of US-responsible vectors for drug delivery applications or the one covered by this review, where US is exploited for the stimulation of piezoelectric nanomaterials for generating localized electric fields, as described in the following sections.

### Interaction between Piezoelectric Nanomaterials and Ultrasound Waves

US waves can be exploited to mechanically activate the piezoelectric nanoparticles, thus locally generating electrical charges thanks to the direct piezoelectric effect. However, the underlying physics of this interaction is not yet entirely clear.

In this regard, only a few basic modeling attempts describing the interaction between mechanical waves and piezoelectric particles have been proposed. An analytical model was developed by Marino *et al.*(55) As shown in the following equation, the voltage generated at the surface (φ) resulted linearly proportional to the radius *R* of the spherical particle and the pressure of the US wave (*P*_US_):
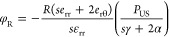
6where *e*_rr_ and *e*_rθ_ are piezoelectric coefficients, ε_rr_ the dielectric constant of the particle, and *s*, γ, and α other material-related parameters. The voltage generated by a single BTNP (diameter: 300 nm) under a US stimulus of 0.8 W/cm^2^ was estimated to be around 0.19 mV. With the same aim, finite element model (FEM) simulations have also been recently proposed using multiphysics software. Zhao *et al.* estimated a generated potential of up to 20 mV by considering a carbon-BTNP under US exposure and in the presence of cavitating bubbles.^56^ Zhu *et al.* found 0.45 V as the maximum piezopotential generated by a cubic barium titanate nanocrystal (size: ∼110 nm) subjected to a very high acoustic pressure (*P*_US_ = 10^8^ Pa).^57^

## Engineering Biological Processes

Table 2 reports the main results achieved in the state-of-the-art using piezoelectric nanotransducers and US stimulation to produce bioeffects on cells.

**Table 2:** Main Results Achieved *In Vitro* Using Piezoelectric Nanomaterials Triggered by Ultrasound Waves, Exploited to Trigger Beneficial Bioeffects on Different Cell Types^a^

reference	nanomaterial type and dimensions	ultrasound parameters	cell type	nanomaterial intra/extracellular location	biological effects observed due to the combination of piezo-nanomaterials and ultrasound
Ciofani *et al.*^58^	BNNTs (*L* = 200–600 nm, *D* = 50 nm) with glycol chitosan coating	*f* = 40 kHz, *P*_w_ = 20 W, *t* = 5 s, four times a day for 9 days (Bransonic sonicator 2510)	rat neuronal-like (PC12) and human neuroblastoma cells (SH-SY5Y)	internalized (cytoplasmic vesicles)	enhancement of neurite elongation (Ca^2+^ fluxes involved)
Ricotti *et al.*^59^	BNNTs (*L* = 200–600 nm, *D* = 50 nm) with glycol chitosan coating	*f* = 40 kHz, *P*_w_ = 20 W, *t* = 10 s, once a day for 7 days (Bransonic sonicator 2510)	murine myoblasts (C2C12)	internalized (early and late endosomes)	overexpression of myogenin, muscle LIM protein, α-actinin, myosin heavy chain (MHC)-IId-x, MHC-IIa, MHC-IIb, and perinatal MHC, at the gene level; production of longer and wider multinucleated myotubes; increase of electrical functionality
Danti *et al.*^60^	BNNTs (*L* < 500 nm, *D* = 40–70 nm) with poly-l-lysine coating	*f* = 40 kHz, *P*_w_ = 20 W, *t* = 5 s, three times a day for 7 days (Bransonic sonicator 2510)	primary human osteoblasts (hOBs)	internalized (membranal vesicles)	enhanced osteopontin expression, osteocalcin production and Ca^2+^ secretion
Ricotti *et al.*^61^	BNNTs (*L* = 200–600 nm, *D* = 50 nm) with glycol chitosan coating	*f* = 40 kHz, *P*_w_ = 20 W, *t* = 5 s, two times a day for 80 h (Bransonic sonicator 2510)	normal human dermal fibroblasts (nHDFs)	internalized	increased F/G-actin ratio and production of thicker stress fibers; enhanced activation of Cdc42, a protein of the Rho family of small GTPases, involved in actin nucleation and polymerization
Marino et al.^55^	BTNPs (300 nm tetragonal crystal) with gum Arabic coating	*f* = 1 MHz, *I* = 0.1–0.8 W/cm^2^, *t* = 5 s (Sonitron GTS sonoporation system)	human neuroblastoma-derived cells (SH-SY5Y)	close to the plasma membrane	activation of voltage-gated Ca^2+^ channels (Cd^2+^) and voltage-gated Na^+^ channels (TTX); intracellular response in terms of Ca^2+^ and Na^+^ fluxes
Marino *et al.*^62^	BTNPs (300 nm tetragonal crystal) embedded in Ormocomp resist	*f* = 1 MHz, *I* = 0.8 W/cm^2^, *t* = 5 s, every 4 h, three times a day for 3 days (Sonitron GTS Sonoporation System)	osteoblast-like cells (SaOS-2)	close to the plasma membrane	enhanced expression of collagen type 1 (COLI) protein (marker up-regulated during osteogenesis) and lower expression of Ki-67 (marker expressed in proliferating cells)
Genchi *et al.*^63^	BTNPs (300 nm tetragonal crystal) in P(VDF-TrFE) film	*f* = 1 MHz, *I* = 1 W/cm^2^, BR = 100 Hz, *t* = 5 s (Sonopore KTAC 4000-KP-S20 probe)	human neuroblastoma-derived cells (SH-SY5Y)	close to the plasma membrane	enhancement of Ca^2+^ transients, β3-tubulin positive cells and neurite lengths
Cafarelli *et al.*^64^	BTNPs (100 nm) with glycol chitosan coating, embedded in a polydimethylsiloxane matrix	*f* = 1 MHz, *I* = 0.2–1.6 W/cm^2^, DC = 20%, BR = 1 kHz, *t* = 3 min (Ultran unfocused transducer)	human dermal fibroblasts (nHDFs)	external to the cells	enhanced proliferation, evaluated through DNA quantification
Genchi *et al.*^65^	BNNTs (*L* = 1 μm, *D* = 10 nm) embedded in a P(VDF-TrFE) film	*f* = 1 MHz, *I* = 1 W/cm^2^, *t* = 10 s, two times a day for 7 days (Sonopore KTAC 4000 device, KP-S20 probe)	human osteosarcoma-derived cells (SaOS-2)	close to the plasma membrane	enhancement of Alpl and Col1a1 proteins (markers of osteoblast differentiation)
Marino *et al.*^66^	BTNPs (300 nm tetragonal crystal) functionalized with anti-HER2 antibody	*f* = 1 MHz, *I* = 0.2–1.0 W/cm^2^, DC = 10%, BR = 0.5 Hz, 1 h a day for 4 days (Sonopore KTAC 4000 device, KP-S20 probe)	breast cancer cells (SK-BR-3)	mostly close to the plasma membrane; scarce nanoparticle internalization	decrease of cell culture metabolism; down-regulation of the expression of Ki-67 proliferative marker; up-regulation of the expression of KCNJ6 (encoding for Kir3.2); increase of intracellular Ca^2+^ levels
Rojas *et al.*^67^	BTNPs (300 nm tetragonal crystal) with gum Arabic coating	*f* = 1 MHz, *I* = 1.0 W/cm^2^, DC = 50%, BR = 0.5 Hz, *t* = 3 min (Sonopore KTAC 4000 device, KP-S20 probe)	primary rat cultures of cortical and hippocampal neurons	close to the plasma membrane (no significant internalization)	enhancement of the mean network firing rate, with almost complete recovery to the original baseline
Chen *et al.*^68^	barium titanate (*D* < 100 nm, cubic crystal) with DSPE-PEG-5000 coating	*f* = 500 kHz, *P*_US_ = 2 kPa, 10 s (Olympus focused transducer)	primary rat cortex neurons	adsorbed on cell membranes	enhancement of spike number and calcium transients with a recovery time of 5 s
Marino *et al.*^69^	BTNPs (300 nm tetragonal crystal) functionalized with anti-TfR antibody	*f* = 1 MHz, *I* = 0.2–1.0 W/cm^2^, DC = 10%, BR = 0.5 Hz, 1 h per day for 4 days (Sonopore KTAC 4000 device, KP-S20 probe)	U87 glioblastoma cells	close to the plasma membrane. In smaller quantities internalized in the cell body	down-regulation of the nuclear proliferation Ki-67 marker; increase of intracellular Ca^2+^ levels
Ma *et al.*^70^	nylon-11 nanoparticles (50 nm)	not reported	dental pulp stem cells (DPSCs)	internalized (endocytosed into the cytoplasm)	enhancement of the Ca^2+^ ions influx; up-regulation of osteopontin (OPN) and osteocalcin (OCN), markers of osteogenic differentiation
Shuai *et al.*^71^	BTNPs (tetragonal crystal) functionalized with polydopamine in a PVDF scaffold	*f* = 100 Hz, *I* = 0.8 W/cm^2^, *t* = 10 s, three times a day (ultrasonic bath)	human osteosarcoma-derived cells (MG-63)	external to the cells (particles embedded into the scaffold)	promotion of cell proliferation; higher alkaline phosphatase (ALP) activity, index of cell differentiation
Zhao *et al.*^56^	BTNPs with carbon shell (*D* = 66 ± 10 nm)	*f* = 1 MHz, *I* = 0.64 W/cm^2^, *t* = 5 min, for 7 days	rat neuronal-like cells (PC12) and wild type AB strain zebrafish (*Danio rerio*)	internalized (endocytic vesicles or endosomes)	enhancement of Ca^2+^ influx through EMF-mediated plasma membrane depolarization; up-regulation of synaptophysin and tyrosine hydroxylase, indicators of synaptic plasticity; change in the spontaneous coiling behavior and activity of zebrafish
Shuai *et al.*^72^	BTNPs (*D* = 200 nm) functionalized with polydopamine and Ag nanoparticles in a PVDF scaffold	*f* = 100 Hz, *I* = 0.8 W/cm^2^, *t* = 10 s, three times a day (ultrasonic bath)	human osteosarcoma-derived cells (MG-63)	external to the cells (particles incorporated into the scaffold)	promotion of cell proliferation; higher alkaline phosphatases (ALP) activity, index of cell differentiation
Zhu *et al.*^57^	BTNPs (tetragonal crystal, *D* = 110 nm) treated with hydrogen peroxide (H_2_O_2_) and embedded in chitosan gel	*f* = 1.0 MHz, *I* = 1.0 W/cm^2^, DC = 50%, *t* = 1–10 min	mammary murine carcinoma-derived cells (4T1); *in vivo* mice bearing 4T1-tumor xenografts	external to the cells	enhancement of redox reactions (piezocatalytic ^•^OH and ^•^O_2_^–^ generation); decrease of cell viability; severe cellular toxicity; *in vivo* suppression of tumor growth; and down-regulation of Ki-67 proliferative marker
Liu *et al.*^73^	BTNPs (*D* = 58 ± 15 nm, tetragonal crystal) with gum Arabic coating and embedded in *Spirulina platensis* micromotor	*f* = 1 MHz, *I* = 1 W/cm^2^	rat neuronal-like cells (PC12)	external to the cells	enhancement of neurite elongation, activation of voltage-dependent Ca^2+^ channels, and adenylyl cyclase pathway

a BNNTs = boron nitride nanotubes, BTNPs = barium titanate nanoparticles, *f* = frequency, *P*_w_ = output power, *t* = stimulation duration, *I* = intensity, *P*_US_ = pressure, BR = burst rate, DC = duty cycle, *D* = diameter, *L* = length, DSPE-PEG-5000 = 1,2-distearoyl-*sn*-glycero-3-phosphoethanolamine-*N-*[methoxy (polyethylene glycol)-5000, EMF = electromagnetic field, PVDF = polyvinylidene fluoride, P(VDF-TrFE) = poly(vinylidene fluoride-trifluoroethylene.

### Stimulation of Electrically Excitable Cells for Neuromodulation Purposes

Electric fields can transiently modulate neural activity in the central and peripheral nervous system by directly acting on neural membrane depolarization and on the threshold potential, which leads to cell excitation or inhibition. Deep brain stimulation has provided clear benefits for patients affected by various neurologic conditions^74^ (*e.g.*, essential tremor, dystonia, pain, Parkinson’s disease). However, such a therapeutic strategy has the severe limitation of relying on surgically implanted electrodes. Focused US has been also proposed as a tool to noninvasively modulate neural activity even in deep regions of the brain.^75^ In fact, mechanical waves can interfere with neuron depolarization through different intracellular biological pathways triggered by the mechanical deformation of the cell membrane. However, this recent approach needs further developments for safe and efficient use *in vivo*.^76^

In this context, the possibility to remotely and safely deliver electrical cues to excitable cells by taking advantage of US-responsive piezoelectric nanoparticles without the need for implanted electrodes might have a tremendous impact.^77^

**Figure 3:**
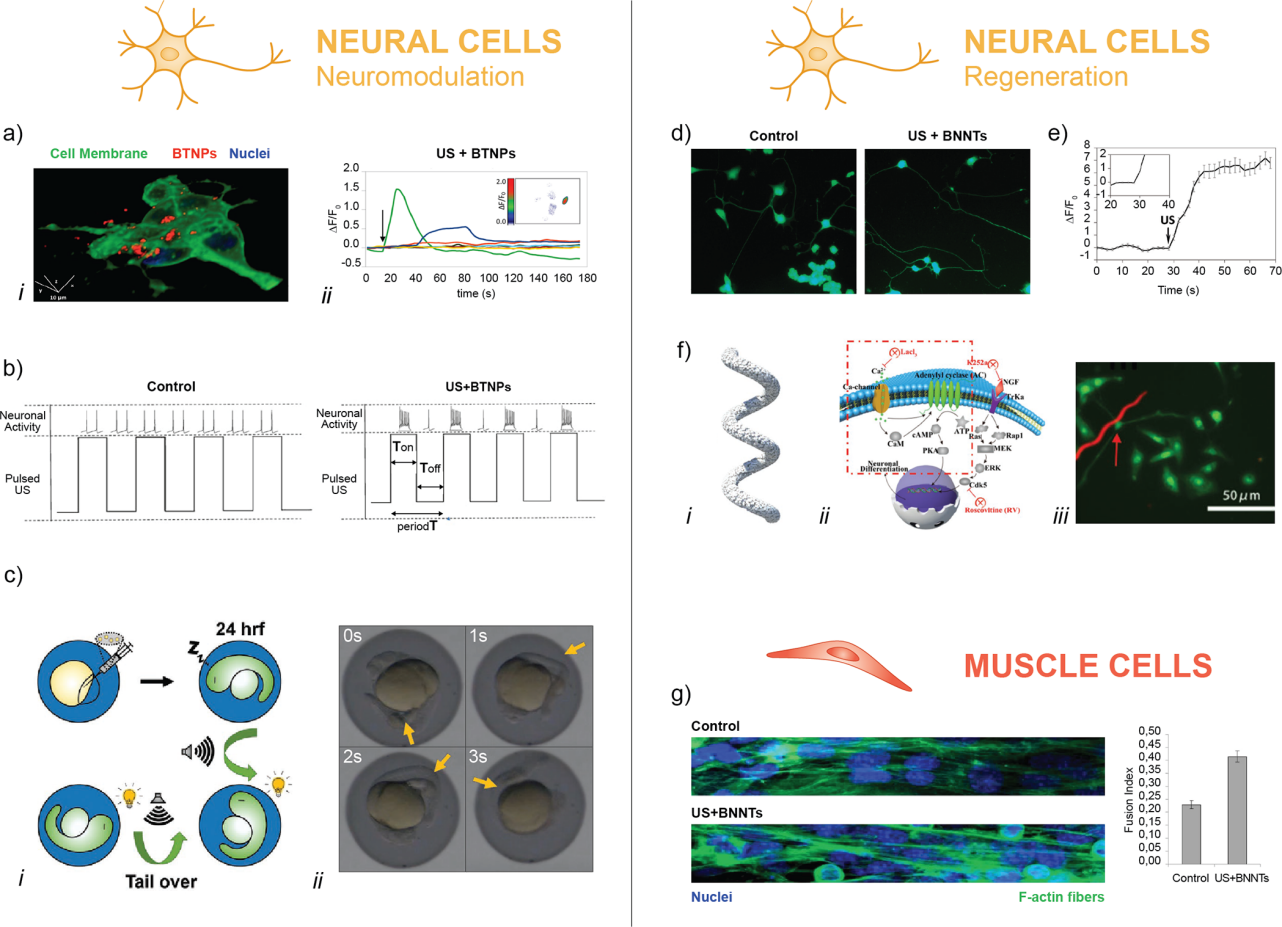
Representative images of the main findings achieved by applying the “US-activated piezoelectric nanoparticle stimulation” paradigm to electrically excitable cells. (a) Barium titanate nanoparticles (BTNPs) internalized within SH-SY5Y-derived neurons (*i*); when ultrasound (US) is applied, a significantly higher calcium flux is detected (*ii*). Images reproduced from ref (55). Copyright 2015 American Chemical Society. (b) Enhancement of the firing rate in neurons provided with BTNPs and stimulated with US.^67^ Images reproduced with permission from ref (83). Copyright 2020 Springer Nature. (c) Depiction of the experimental procedure (*i*) and photos showing changes in the spontaneous coiling behavior on a zebrafish embryo induced by BTNPs and US (*ii*). Images adapted with permission from ref (56). Copyright 2020 John Wiley and Sons. (d) Enhancement of neurite elongation in PC12 cells internalizing BNNTs and stimulated with US. Images reproduced from ref (58). Copyright 2010 American Chemical Society. (e) US+BTNPs enhances Ca^2+^ transients in SH-SY5Y cells. Image reproduced with permission from ref (63). Copyright 2016 John Wiley and Sons. (f) US stimulation of *S. platentis* with BTNPs (*i*) triggers different intracellular pathways affecting PC12 cell differentiation (*ii*) and mediating neurite outgrowth (*iii*). Images reproduced with permission from ref (73). Copyright 2020 John Wiley and Sons. (g) C2C12 cells internalizing BNNTs and stimulated with US receive a boost to form multinucleated myotubes featured by a higher fusion index (index of skeletal muscle tissue maturity). Images reprinted with permission under a Creative Commons Attribution License from ref (59). Copyright 2013 Ricotti *et al.*

A direct proof of neural activation in response to US stimulation of internalized piezoelectric nanoparticles was observed on SH-SY5Y cells^55^ (Figure 3a) and primary neurons.^67^ US waves were used in these works to activate BTNPs, mostly located on the plasma membranes of the neural cells after 24 h of incubation. In both works, significant neural activation was detected only when adopting piezoelectric noncentrosymmetric BTNPs (with tetragonal crystal structure), whereas no cell excitation was detected using nonpiezoelectric centrosymmetric BTNPs (with cubic crystal structure). These results indicated that neural activation was actually mediated by the material piezoelectricity, and permitted excluding the involvement of other nonspecific phenomena (*e.g.*, thermal or mechanical ones). An enhancement of the mean network firing rate (*i.e.*, the average number of the detected spikes per second) due to US stimulation and piezoactivity was demonstrated^67^ (Figure 3b). The process also proved to be reversible: neuron response can be fully recovered a few seconds after switching off the stimulus.^68^ These works revealed the safe and reversible nature of the “US-activated piezoelectric nanoparticle stimulation” paradigm and highlighted a correlation between the US power intensity and the probability of activating the BTNP-incubated neurons.

Recently, experimental evidence on an *in vivo* model (*i.e.*, the zebrafish embryo, *Danio rerio*) showed the ability of the above-mentioned stimulation paradigm to modulate neural plasticity and recover degenerated dopamine neurons,^56^ highlighting a possible impact in the treatment of neurodegenerative diseases. The authors found that US-activated BTNPs provided with a carbon shell induced an up-regulation of tyrosine hydroxylase and synaptophysin, key markers for dopamine neuron regeneration and synaptic plasticity, which reflected in an alteration of the spontaneous coiling behavior of the zebrafish embryos (Figure 3c).

### Stimulation of Electrically Excitable Cells for Regenerative Purposes

Electrical stimulation has been widely exploited in tissue engineering and regenerative medicine as a tool for promoting the differentiation of electrically responsive cells toward mature phenotypes. Usually, this kind of stimulus is provided to cells through simple electrodes or microelectrode arrays integrated into the cell/tissue culture system.

The “US-activated piezoelectric nanoparticle stimulation” paradigm has the potential of generating electric fields not only in close proximity of the cells but even inside them. Experimental proof in this domain was obtained in 2010 on an *in vitro* neural-like cell model based on nerve growth-factor-treated PC12 pheochromocytoma cells.^58^ In this work, Ciofani *et al.* observed an enhanced morphological differentiation of the PC12 neural-like cells (Figure 3d) in response to the combined treatment with US and boron nitride nanotubes (BNNTs, internalized within cells) compared to that in the control cultures (*i.e.*, nontreated cells, cell exposed to the bare US stimulation without piezoparticles, and cells incubated with BNNTs but not stimulated with US). Experiments in the presence of the LaCl_3_ inhibitor suggested the involvement of Ca^2+^ influx in mediating this effect. This pioneering work enabled a series of subsequent investigations on different neural cell/progenitor models, including dorsal root ganglion neurons,^78^ human neural stem/progenitor cells,^79^ rat spinal cord neurons,^80^ SH-SY5Y-derived neurons^63^ (Figure 3e), and again PC12 neural-like cells,^81^ although in some of these studies, piezoelectric films, rather than nanomaterials, were investigated.

These independent investigations commonly reported an enhanced neural differentiation in terms of neurite elongation, neuritogenesis, and expression of the β3-tubulin molecular marker.

Recently, exciting applications of the piezostimulation on neural differentiation and regeneration have been proposed. In the work of Liu *et al.*, magnetic/piezoelectric micromotors have been fabricated *via* integration of the *S. platensis* with Fe_3_O_4_ magnetic nanoparticles and BTNPs^73^ (Figure 3f). These micromotors have been used to precisely target single stem cells under a rotating magnetic field and boost their differentiation by converting US energy into electrical cues due to the direct piezoelectric effect.

Compared to neural cells, only a few works have been devoted to the piezostimulation of muscle cells. Ricotti *et al.* demonstrated the possibility to exploit the “US-activated piezoelectric nanoparticle stimulation” paradigm for boosting the differentiation of skeletal muscle cells *in vitro*(59) (Figure 3g). In this work, a synergy between electrical (piezomediated), chemical (due to a coculture with fibroblasts), and mechano-topographical (due to the substrate features) stimuli permitted achieving a mature engineered tissue. An evident myogenic potential of piezoelectric-based stimulation was subsequently confirmed in an interesting work of Yoon *et al.*, in which human umbilical cord blood mesenchymal stem cells were cultured on a stretchable thermosensitive piezoelectric substrate provided with aligned ZnO nanorods and then subjected to mechanoelectrical cues by repetitive stretching and bending cycles.^82^ The thermosensitivity of the proposed scaffold allowed the subsequent detachment of the differentiated cell sheet fragments and their injection into injured mouse skeletal muscle. Enhanced muscle regeneration was demonstrated *in vivo*.

### Stimulation of Non-electrically Excitable Cells for Regenerative Purposes

**Figure 4:**
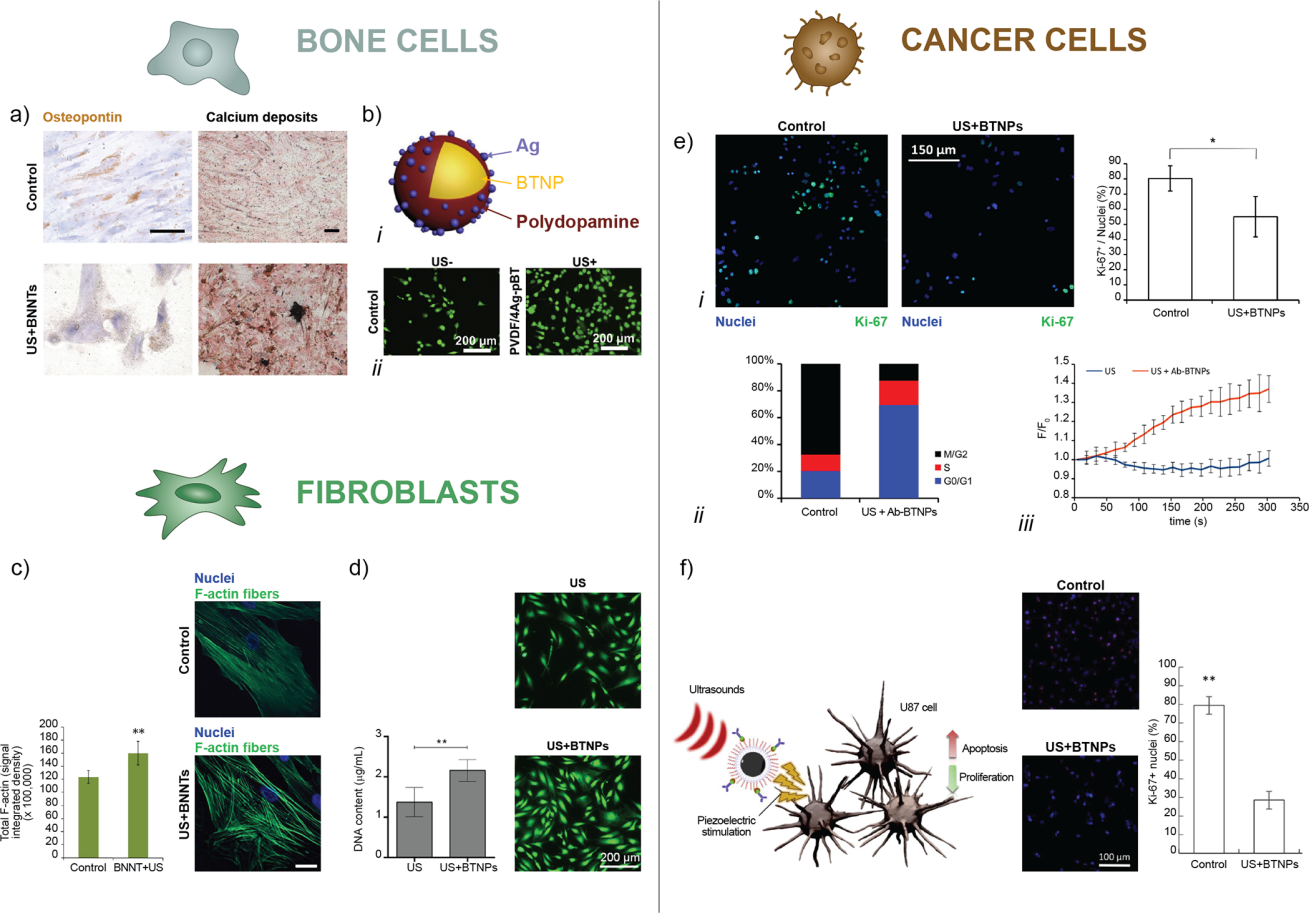
Representative images of the main findings achieved by applying the “piezonanoparticles + US” paradigm to non-electrically excitable cells. (a) BNNTs in combination with US up-regulate calcium production and osteopontin expression in osteoblasts. Image reproduced with permission from ref (60). Copyright 2013 IOP Publishing. (b) Ag-conjugated barium titanate nanoparticles (BTNPs) (*i*) stimulated with US enhance the proliferation of osteosarcoma-derived cells (*ii*). Images adapted with permission from ref (72). Copyright 2020 Elsevier. (c) F-actin overexpression in dermal fibroblasts triggered by US + BNNTs. Images reproduced with permission from ref (61). Copyright 2014 Springer Nature. (d) US enhances proliferation of fibroblasts in the presence of BTNPs, embedded in a scaffold. Images reproduced with permission from ref (64). Copyright 2017 Elsevier. (e) Combination of US and BTNPs down-regulates the Ki-67 proliferative marker in breast cancer cells (*i*), induces the arrest of the cell cycle in G0/G1 phases (*ii*), and increases the intracellular concentration of calcium (*iii*). Images reprinted with permission under a Creative Commons CC BY License from ref (66). Copyright 2018 Springer Nature. (f) US-induced piezoelectric treatment induces cell apoptosis and decreases proliferation in glioblastoma cells. Images adapted with permission from ref (69). Copyright 2019 Elsevier.

Neurons and muscle cells are defined as electrically excitable cells due to their stereotyped response to electric cues (*e.g.*, action potential and contraction). However, many other cell types express voltage-sensitive channels and are indeed sensitive to electrical stimulations (*e.g.*, osteoblasts, chondroblasts, fibroblasts, stem cells, and cancer cells).^84^ Moreover, it is worth mentioning that the intrinsic piezoelectricity of collagen and bone tissue appears to play a key role in regulating tissue regeneration through mechanoelectrical transduction.^16^ The “US-activated piezoelectric nanoparticle stimulation” paradigm has been exploited *in vitro* to promote osteoblast differentiation/maturation at both the gene and the protein level. In this regard, Danti *et al.* demonstrated the possibility to exploit BNNTs and low-frequency US to trigger the differentiation process in primary human hosteoblasts,^60^ obtaining an overexpression of osteopontin and osteocalcin proteins (Figure 4a). Subsequent works have also evidenced an enhancement of the expression of collagen type 1 (COL1A1)^62,65^ and a down-regulation of the Ki-67 protein.^62^ Recently, Shuai *et al.* proposed “strawberry-like” silver-BTNPs incorporated in a PVDF scaffold to promote human osteoblast-like cell proliferation and differentiation when stimulated by US waves (Figure 4b).^72^

Chondroblasts and chondrocytes also demonstrated responsiveness, although these results were obtained not using piezoelectric nanomaterials combined with US but ultrasonically active coverslips^85^ and polymeric patches doped with piezoelectric nanopowder but without US stimulation.^86^ Grounded on these results, researchers have envisioned the use of the piezoelectric stimulation approach with US to remotely treat osteoporosis and osteoarthritis and, more generally, to promote regeneration of the skeletal system.^87,88^

Another interesting behavior has been observed in fibroblasts. In 2014, BNNTs were internalized in human dermal fibroblasts, and then US stimulation was provided. This produced a marked increase of the F/G-actin ratio with respect to the nonstimulated controls, suggesting that such an intracellular stimulation promoted actin polymerization pathways. This was also confirmed by the production of thicker stress fibers in the cell cytoskeleton and by increased activation of Cdc42, a protein involved in actin nucleation and polymerization^61^ (Figure 4c). In 2017, Cafarelli *et al.* precisely correlated the bioeffect obtained (*i.e.*, human fibroblasts proliferation, Figure 4d) with the intensity of the US provided, stimulating a polydimethylsiloxane substrate doped with BTNPs. The US dose delivered to cells was precisely controlled (1 MHz, 800 mW/cm^2^, 20%, duty cycle, 1 kHz burst rate), also calculating the attenuation produced by the piezoelectric scaffold, thus enabling a reliable comparison of these biological results with the ones obtained with the same US dose but without the piezoelectric scaffold interposed in the acoustic path.^64^

### Therapeutic Bioeffects on Cancer Cells

It is well-known from the literature that a mild electrical stimulation is effective in inhibiting cancer cell proliferation, as an alternative to other (more traditional) approaches.^89,90^ Electrical stimulation also enhances the therapeutic effect of chemotherapy in brain cancer.^91^ These strategies have been approved by the Food and Drug Administration for the treatment of glioblastoma multiforme,^92^ and trials are ongoing for the treatment of ovarian cancer and pancreatic adenocarcinoma.^93^ Low-intensity electrical cues are indeed able not only to affect cancer cell proliferation without the use of any drugs/chemicals but also to reduce multidrug resistance phenomena. Furthermore, cells originated from abnormal mitosis events due to the chronic electric stimulations result unable to proliferate.^90^

A significant drawback of such an approach is related to undesired stimulation of healthy cells, whose proliferation can be affected by electrical cues. For this reason, local and well-targeted delivery of electric cues specifically to cancer cells would be highly desirable. The “US-activated piezoelectric nanoparticle stimulation” paradigm would constitute a promising tool to solve such an undesired effect. Recent findings reported the successful remote stimulation of different cancer cell types by the synergic exploitation of inorganic piezoelectric nanoparticles combined with US. *In vitro* studies with BTNPs showed that chronic piezoelectric stimulation arrests cancer cell cycle in G_0_/G_1_ phases by interfering with Ca^2+^ homeostasis and up-regulating the expression of the gene encoding for Kir3.2 inward rectifier K^+^ channels. Moreover, the organization of cytoskeletal elements mediating cell mitosis is affected. Anticancer effects have been proven both on breast cancer cells^66^ (Figure 4e) and glioblastoma multiforme cells^69^ (Figure 4f). Recently, Racca *et al.* demonstrated efficient killing capability on cervical adenocarcinoma cells of ZnO nanocrystals combined with high-energy US shock waves, even if the role of the piezoelectric effect on cell death remained unclear in this study.^94^ A recent *in vivo* experiment also demonstrated the ability of the US-BTNPs combination to generate toxic reactive oxygen species (ROS), thus down-regulating the Ki-67 proliferative marker and showing a piezo catalytic tumor eradication potential.^57^

## Outlook

**Figure 5:**
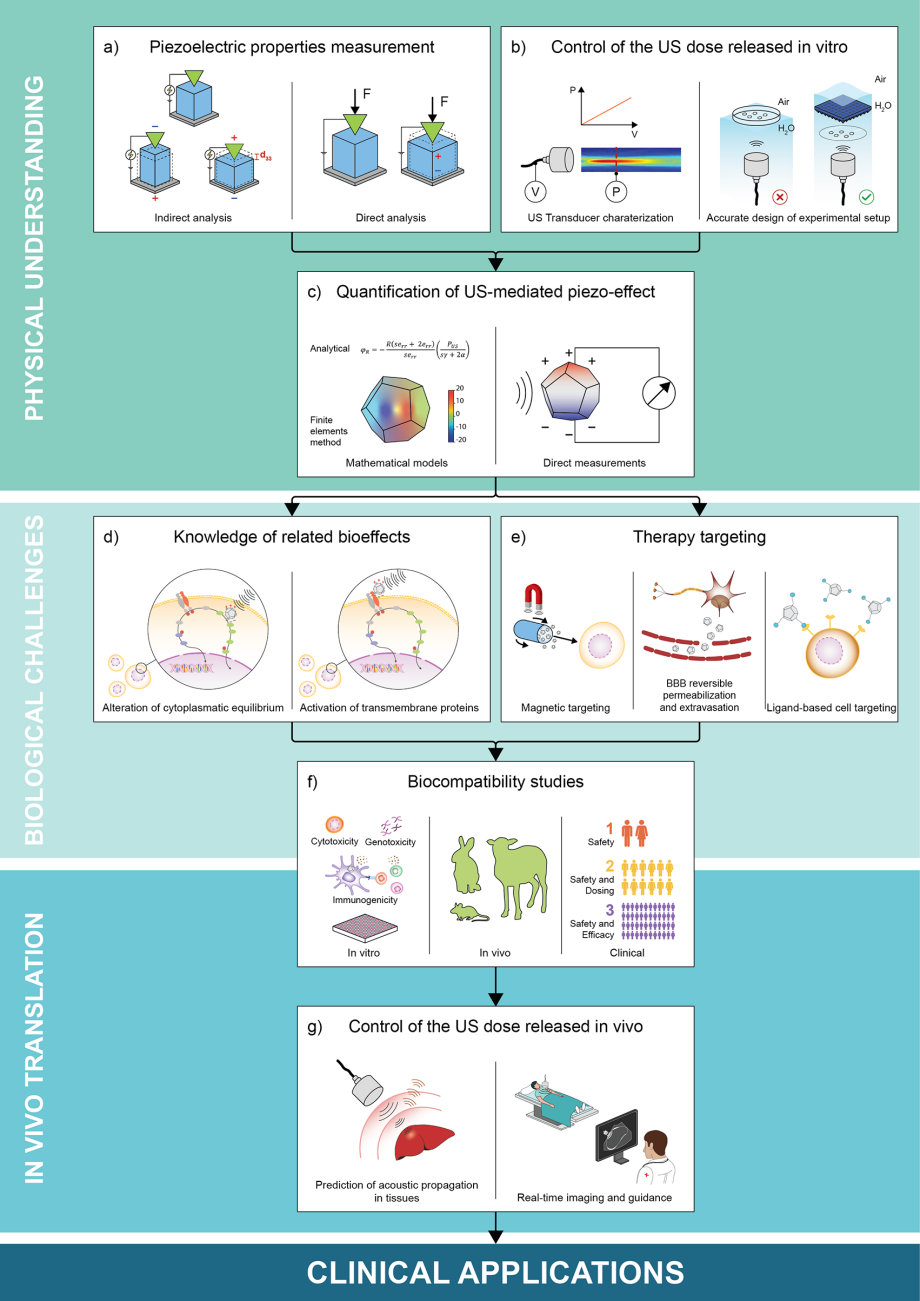
Scheme of the key aspects to be addressed to foster clinical acceptance of the “US-activated piezoelectric nanoparticle stimulation” paradigm. The identified route includes the following substeps. (a) *Piezoelectric properties measurement*: accurate quantification of the piezoelectric coefficients plays a crucial role in the experimental design phase and material selection. (b) *Control of the US dose released in vitro*: the use of dose-controlled stimulation systems enables a precise correlation between the effective US dose and the biological findings. (c) *Quantification of US-mediated piezo effect*: the interaction between US waves and piezoelectric particles needs to be further explored for a better understanding of the underlying phenomenon. (d) *Knowledge of related bioeffects*: the activated cellular mechanisms need to be elucidated more in-depth. (e) *Therapy targeting*: new approaches for enhancing the spatial localization of the therapy delivery result crucial for a targeted *in vivo* use. (f) *Biocompatibility studies*: careful analyses about nanoparticles biosafety have to be accomplished before their use in the clinics. (g) *Control of the US dose released in vivo*: US must be correctly tuned in order to reach the target *in vivo* with the desired dose.

As depicted in Figure 5, the achievement of effective or even game-changing clinical applications of the “US-activated piezoelectric nanoparticle stimulation” paradigm should pass through a series of steps. They involve: (*i*) “physical understanding” of the phenomenon and the properties of materials and energy source used; (*ii*) “biological challenges”, referring to fully unveiling the intracellular processes engineered by this stimulation, as well as to implementing a targeted therapy; (*iii*) “*in vivo* translation”, which involves carefully checking the bio- and immunocompatibility of the nanoparticles delivered in the body but also controlling and bringing the optimal US doses in the complex and heterogeneous *in vivo* scenarios. Some of the mentioned blocks have been addressed in the past decade, and many discoveries have been made. However, other blocks have been scarcely explored or even entirely neglected so far in the state-of-the-art. Thus, they constitute crucial open challenges that must be addressed to make the “US-activated piezoelectric nanoparticle stimulation” paradigm useful to patients. In the next sections, each block will be described and the authors’ perspective on its possible future evolution will be given.

### Piezoelectric Properties Measurement

Accurate measurement of nanomaterial piezoelectric properties (Figure 5a) is a complicated process, but it is important for selecting the most suitable material for a specific biomedical application, as well as for supporting accurate analytical or numerical models. This step, if correctly performed, can actually help in correctly understanding and quantifying the physics of the piezo effects mediated by US. In 1992, piezoresponse force microscopy (PFM) was found to be a promising method to detect and quantify the piezoelectric properties of nanomaterials.^95^ This technique has been widely explored recently, becoming a standard characterization tool at the micro- and nanoscale.^96^ PFM is a particular modality of scanning probe microscopy that uses an alternating current (AC) voltage to infer the material piezoelectric coefficients. The standard PFM modality exploits the indirect piezoelectric effect, whereas only recently there have been advancements in the adoption of the direct effect to extrapolate electrical signals from nanomaterials.^97^

PFM is widely used nowadays because of its high resolution, nondestructive imaging capability, and the possibility to measure the local piezoelectric activity even in nanomaterials with complex geometries. In the authors’ opinion, the standard on piezoelectricity elaborated in 1988 by the Institute of Electrical and Electronics Engineers^98^ does not entirely comply with the need for adequately analyzing the piezoelectric activity of nanomaterials, which emerged in the past decade. Indeed, for these materials, some challenges still need to be addressed.

The measurement of the indirect piezoelectric effect through PFM is generally affected by (*i*) electrostatic phenomena between the cantilever and the sample surface and (*ii*) dependence on the resonance frequency of the tip. These issues might lead to misinterpretations and lack of robustness of the measured PFM response, suggesting incorrect amplitude values (and thus incorrect piezoelectric coefficients) or wrong location of the domain boundaries.^99,100^ Adopting stiff cantilevers may partially solve the issue of electrostatic phenomena, but it restricts the analysis only to rather resistant materials (*e.g.*, ceramic ones). A decisive step ahead to mitigate the mentioned drawbacks has been the implementation of the dual AC resonance tracking PFM in commercial instruments to increase sensitivity and avoid dielectric breakdown of materials, thus enhancing the robustness of the acquired data.^101^ In parallel, the recent development of a spectroscopic form of the PFM (*e.g.*, the DataCUBE modality implemented by Bruker) enables the acquisition of a force–distance spectrum at every pixel. Thus, topographical, mechanical, and multidimensional electrical data lead to a broader understanding of the piezoelectric behavior. Meanwhile, it is expected that nondestructive PFM modalities will emerge as valid alternatives to evaluate the piezoelectric activity also in softer materials than the most common ceramics or relatively rigid thermoplastics polymers.^102^

Measuring the direct piezoelectric response has the advantage of being less sensitive to electrostatic phenomena since the electric field is not applied during the measurement. In this way, the piezoelectric features of the material might be detected in a more precise way.^103^ Despite this advantage, a few reports have been published on this kind of detection modality, so far. Indeed, the electronics acquiring the signal is less straightforward to be designed as it must detect a meager amount of current or voltage, especially in nanosized materials, with a higher risk of undesired noise.

The authors believe that many improvements have been brought into this field, from the introduction of PFM to the setting of more advanced methods. To date, we are still not able to define a single and entirely correct approach to measure the piezoelectric coefficients of nanomaterials. Although PFM represents a valuable platform to investigate nanomaterial piezoelectric properties, we are still far from considering it as a part of a standardized evaluation test-bench instrument for industrial applications. A standardized approach will be essential to make PFM measurements carried out in different laboratories consistent and reliable. A protocol for best-practice measurements should be outlined to identify the uncertainty induced by possible variables, such as the use of tips with different features. For this purpose, commercially available piezoelectric samples, such as the lithium niobate, could be used to calibrate the PFM setup. The establishment of a quantitative characterization protocol will guarantee a process of homogenization of the approaches carried out by different laboratories in the next future.

### Control of the US Dose Released *in Vitro*

In any study that attempts to find a quantitative relationship between the US exposure and the observed effect *in vitro*, scientists should measure or at least correctly estimate the exposure dose at the target (where the effects are observed) and report it in a correct manner.^104^ Indeed, although a non-entirely controlled/known US dose at the target can be acceptable in an initial stage when a new phenomenon/effect is discovered, it becomes crucial when looking for the optimal dose and when the biophysical stimulus is engineered to obtain the maximum desired biological effect.^105^

Poorly standardized configurations, lack of proper calibration, and lack of control on US wave reflections/attenuations jeopardize the reliability and the comparison between *in vitro* and *in vivo* studies and even between different *in vitro* experiments carried out through different setup configurations. This obviously slows down the translation of such efforts to clinical reality. The vast majority of the studies reported in Table 2 are affected by such issues: some of them do not report all the US parameters used, and other ones exploited sonication baths, which cause an unpredictable exposure condition since the operator can set only the electric power of the device, but no information about the US dose reaching the samples can be derived or accurately measured. In a few studies, different frequencies and intensities were tested. However, mainly due to missing or limited information about the US source and acoustic propagation within the setup, results may not be comparable and reproducible by other researchers.

A fundamental step required for a controlled US stimulation is an appropriate characterization of the employed transducers, thus to be aware of the pressure map and the relationship between the driving voltage and pressure intensity at the target for each exposure condition^106^ (Figure 5b). This would allow knowing, point-by-point, the exact dose of acoustic energy delivered and would allow setting the most appropriate distance between the US source and the biological target, an aspect that is also often neglected. In fact, positioning the sample too close to the US source, in the so-called “near-field” (*i.e.*, at distances lower than *D*^2^*/*4λ, with *D* = diameter of the transducer and λ = wavelength) leads to extensive fluctuations in the US intensity spatial distribution. It is a good practice to position the sample in the “far-field”, thus guaranteeing a greater uniformity of the US regarding the local intensity distribution.

Another critical issue is the design of the experimental system used for US experiments. Inappropriate setups generate uncertainty in the exposure that for *in vitro* cell stimulation can exceed 700%.^107^ For example, a vast majority of systems currently used in this field^108^ exploit nontransparent materials (*e.g.*, standard plastic Petri dishes) along the US beam path: they reflect part of the energy and may produce some hardly predictable effects, such as standing wave formation.^109^ The experimental setups should be therefore designed and built up, trying to minimize these typical exposure errors. To this purpose, particular attention needs to be devoted to knowing the acoustic properties of materials interacting with the US waves in the stimulation path, thus enabling the correct prediction of the US dose at the target.^64^*In situ* measurements (*e.g.*, through hydrophones) or acoustic simulations (*e.g.*, finite element methods) should also be performed to fully control/predict the dose at the target, giving significant added value to the *in vitro* findings. This would also allow varying the stimulation conditions (*i.e.*, frequency, intensity, duty cycle, stimulation time), verifying the bioeffect produced by each specific dose and thus finding the optimal one in a reliable way.

Additionally, *in situ* measurements of US-induced physical effects (*e.g.*, thermal ones, by means of fine wire thermocouples^105^ or thermal cameras^110^) could help interpret the results by excluding other phenomena that could superimpose with the electrical ones.

### Quantification of US-Mediated Piezo Effect

One of the most significant problems slowing down the translation of piezoelectric stimulation to the clinical reality is the lack of clear knowledge of the underlying physical phenomenon. In fact, the “US-activated piezoelectric nanoparticle stimulation” paradigm is often exploited as a “black box”, showing only its observed effects on the final target (which are significant but do not clarify the full picture).

Except for the few analytical and numerical modeling attempts reported in the “Interaction between Piezoelectric Nanomaterials and Ultrasound Waves” section, a detailed physical explanation of the phenomenon is currently still missing. On the one hand, analytical models are necessarily based on important and sometimes nonrealistic assumptions (*e.g.*, homogeneous, isotropic, and linearly elastic materials, spherical geometry, *etc.*). On the other hand, numerical solutions do not always accurately describe all the relevant physical phenomena of the “nanoworld”.^111^ The required computational resources often constitute a limiting factor.

Above all, a real and correct measurement of the electrical charges generated by piezoelectric nanomaterials when US is applied has not been performed yet. Zhao *et al.* recently fabricated a measurement device for quantifying the generated electrical potential with a sandwich structure composed of two electrodes within which nanoparticles are positioned.^56^ However, in the authors’ opinion, in this kind of configuration, the results are strongly affected by capacitive effects caused by the electrodes subjected to US vibrations.

Correct quantitative data derived from electrical measurements of the piezoparticles subjected to US waves will represent real evidence of this concept and could push the exploitation of this promising approach in the clinics. Bench tests analyzing different experimental conditions, both from the US stimulation side (*e.g.*, using different frequencies, intensities, and field geometries) and from the nanoparticle side (*e.g.*, particle types, crystal structures, dimensions, concentrations) will represent the next step needed toward treatment optimization and consequent medical acceptance. As depicted in Figure 5c, the authors strongly believe that more profound knowledge of the phenomenon, supported by appropriate modeling and quantification of the induced charges when a specific US stimulation is applied, is a crucial step for further developments in the US-mediated piezoelectric stimulation field. These results would also generate greater awareness in future *in vitro* and *in vivo* experiments, facilitating the exploration of different application domains.

### Knowledge of Related Bioeffects

The mechanisms of cell response to piezoelectric stimulation have not been fully elucidated yet. Involved pathways can be affected by multiple factors/conditions, such as the source and protocol of the mechanical stimulation (as mentioned in the previous section), the cell type adopted in the study, and the subcellular localization of the nanomaterial (Figure 5d). The complexity of these investigations is also associated with the difficulty in distinguishing and excluding nonspecific phenomena (*e.g.*, thermal and mechanical ones) that might superimpose with the electrical ones, interfering with the cell behavior and leading to a possible misinterpretation of the results. For this reason, carrying out experiments with multiple control conditions, including the mechanical stimulations without the presence of the piezoelectric nanomaterials, and, when possible, by substituting the piezomaterials with their nonpiezoelectric analogues, is highly desirable.

As reported in the previous sections, some contrasting results were recorded on different cell types. For example, piezoelectric stimulation can promote the proliferation of fibroblasts^64,112^ and macrophages^113^ but instead inhibits the cell cycle progression in different malignant cells, such as breast cancer cells^66^ and glioblastoma multiforme cells.^69^ However, these different cellular responses are only apparently contradictory. Indeed, similarly to piezostimulation, direct electrical stimulation is also known to promote the proliferation of fibroblasts^114^ and inhibit that of cancer cells through cell cycle arrest and mitotic spindle disruption.^89,90^ In this regard, it is worth mentioning that the biochemical pathways triggered by piezoelectric and direct electrical stimulations are typically comparable.

Concerning neurons, drug treatments with specific blockers demonstrated the activation of Ca^2+^ and Na^+^ voltage-gated channels in response to the nanoparticle-mediated piezoelectric stimulations.^55^ An alternative mechanism of neuron activation in response to the voltage generated by nanoparticles might contemplate the redistribution of the divalent ions on the external surface of the plasma membranes and, consequently, an increase of the voltage sensitivity of the voltage-gated channels (*i.e.*, a shift of the activation curves of the voltage-gated channels).^115^ However, it is not yet clear how and if the piezoelectric activation of the neurons can be modulated by tuning US stimulation parameters (*e.g.*, frequency). Finally, the promotion of neural differentiation/maturation under piezoelectric stimulation was demonstrated in independent studies in terms of neurite elongation,^58,63,78,79^ neuritogenesis,^80,81^ and increased expression of the β3-tubulin molecular marker.^63,79^ Interestingly, the neuritogenesis induced by US-driven piezoelectric stimulation is mediated by the cyclic adenosine monophosphate (cAMP)-dependent pathway, an independent mechanism with respect to the well-studied mitogen-activated protein kinases/extracellular signal-regulated kinases (MAPK/ERK) pathway.^81^ Further investigations should be directed to clarify this point. This would help to safely target and remotely activate specific cell populations involved in the regulation of different pathologic conditions, such as the basal ganglia neurons of the direct pathway in patients with Parkinson’s disease, having a tremendous impact on nanomedicine of the future.

Chronic piezoelectric stimulation is known to induce positive effects on the differentiation and maturation of different cell types. Among them, the stimulation of muscle cells through piezoelectric nanomaterials and US would deserve further investigations for clarifying in detail the activation mechanisms and for excluding possible toxic or nonreversible effects in response to chronic stimulation.

Chondrogenic differentiation of mesenchymal stem cells was enhanced by piezostimulation on quartz substrates with US waves; in this case, cell clustering, activation of the *Wnt* signaling, and up-regulation of the SOX9 chondrogenic marker were observed after 3 days of chronic treatment.^85^ This approach also needs further developments: new findings in this research field could have a significant impact on the treatment of degenerative joint diseases, such as osteoarthritis.

Finally, the biological effects of the piezoelectric stimulation are expected to be determined by the subcellular localization of the piezoelectric nanomaterial. Although the piezoelectric stimulation of the plasma membrane and the involvement of the voltage-gated channels have been demonstrated, future investigations should also elucidate the biochemical pathways triggered by the intracellular and intraorganelle stimulations. The functionalization of the piezo-nanomaterials with molecular moieties for targeting specific organelles would represent a preliminary step toward the fine regulation of the cellular behavior. Moreover, a systematic comparison of the signaling activated by scaffolds (extracellular localization) or internalized nanoparticles (intracellular localization) may shed light on the different stimulation mechanisms and the associated bioeffects.

### Therapy Targeting

Recently, a thorough review of the delivery of nanoparticles to tumors has concluded that the median delivery efficiency of these nanostructures to target sites is only 0.7%.^116^ This highlights that alternative strategies are essential to accomplish a successful delivery of nanoparticles to the target tissues^117^ (Figure 5e). Understanding the journey that a nanomaterial must undertake until reaching the targeted tissue is crucial. The main hurdles of this journey are the barriers, which can be divided into the following levels: (*i*) the organ barrier level (*e.g.*, reticuloendothelial systems of liver and spleen, protein corona); (*ii*) the suborgan barrier level (vascularization of the tissue, variations in the populations, and distribution of immune cells within a tissue); and (*iii*) the subcellular level (endosomes). A primary step in designing a nanoparticle-based therapy system consists of using libraries of nanoparticles, evaluating their interactions *in vitro* and *in vivo*, with specific affected tissues, as a function of their shape, size, and surface chemistry. The biology of the target site will also determine the chemistries necessary not only to overcome the barriers but also to maximize the required interaction for a treatment. These studies are urgently required, especially in the area of piezoelectric nanomaterials. Based on experimental data, the use of computational approaches may aid in the quest for finding those piezoelectric nanoarchitectures most suitable for treating a specific disease. It is worth mentioning that the use of piezoelectric nanomaterials for delivering electric fields and electrostimulating cells at target sites is a different problem than using nanoparticles to carry a therapeutic agent. Nevertheless, piezoelectric nanomaterials have been much less investigated than other nanoparticle systems such as those made of gold or silica. Research efforts toward the functionalization and bioderivatization of piezoelectric surfaces are timely, as mentioned in the previous section “Piezoelectric Nanomaterials”.

Regarding suborgan barriers, it has been demonstrated that physical triggering strategies (*e.g.*, magnetic and electric fields, light, and US) can temporarily and reversibly modify the permeability of cell membranes, thus consequently enabling targeted delivery of therapeutic agents, cells, or biomolecules.^118,119^ In this context, focused US under magnetic resonance imaging (MRI) guidance and in conjunction with intravenously injected microbubbles has emerged as a powerful technology to transiently and safely open the blood–brain barrier (BBB),^120^ a highly specialized vascular structure that strongly limits the extravasation in the brain of the vast majority of substances circulating in the blood. The authors strongly believe that this approach could be applied in the future to precisely deliver piezoelectric nanoparticles across the BBB into the central nervous system pathological areas. Further investigations should be directed to ensure the safety of the overall process and characterize the diffusion of the piezo-nanoparticles in the brain parenchyma.

A different targeting strategy may consist of providing motion capabilities to the particles. This strategy not only would aid in overcoming barriers but also would enable maximizing the accumulation of nanoparticles in shorter times. In this vein, a considerable amount of research has been devoted to developing motile micro- and nanostructures, known as small-scale motors, capable of swimming in several fluid environments through different energy sources. With their motility features, these devices could navigate the complex vasculature of the body and efficiently deliver drugs to specific targets.^121^ Among the motors’ family, we can distinguish small-scale robots, which are micro- and nanodevices that can swim thanks to external sources of energy, such as magnetic fields, US, light, or combinations. Their speed, directionality, and on/off motion can be readily controlled by modulating or switching on/off the applied source of energy.^121^ As mentioned previously, US is widely used in medical imaging and diagnosis. The features of US-based imaging technologies could be combined with the possibility of acoustically controlling the motion and actuation of US-responsive piezoelectric small-scale architectures. The resulting integrated small-scale robots with electric-field delivery capabilities could be used for a plethora of biomedical applications. Acoustic manipulation of small robots can be achieved in several ways. Acoustic manipulation through stationary waves is quite attractive for *in vitro* experimentation.^122^ However, this strategy is not suitable for *in vivo* applications because forming a predictable standing wave pattern in a living organism is challenging. The use of traveling waves is more appealing for biomedical applications. By carefully selecting materials with different acoustic impedance, it is possible to generate propagating waves in small-scale structures such as segmented nanowires. Ahmed *et al.*, for instance, demonstrated the eukaryotic cell-like propulsion of hinged nanoswimmers consisting of a metallic head linked using a soft polymeric hinge to a flexible polymeric tail.^123^ Another interesting strategy to induce motion in small-scale structures is by introducing cavities with trapped air bubbles. When an acoustic field is applied, the microstreaming generated in the surrounding fluid generates the propulsion force acting on the microrobot.^124^

In the authors’ opinion, in addition to the need for systematic *in vitro* and *in vivo* studies on the biocompatibility and cytotoxicity of piezoelectric and ferroelectric nanoarchitectures—see next section—ground-breaking approaches for concentrating piezoelectric nanostructures at the target sites must be further investigated. We believe that endowing suitable functionalization to the structures as well as providing them the ability to move through biologically relevant fluids will not only facilitate the use of piezoelectric materials in the biomedical arena but will also accelerate their translation from the bench to the clinics.

### Biocompatibility Studies

Each time a new nanomaterial is envisioned for a biomedical application, several concerns arise about biosafety and toxicity. As depicted in Figure 5f, the route toward clinical translation is very long and challenging, and each step forward requires extreme caution in evaluating any potential detrimental effect of the proposed nanoparticle.^125^

Concerning piezoelectric nanomaterials, the evaluation of their safety and biocompatibility is of paramount importance since the very early stage of their investigation. This is particularly relevant if we consider that, quite often, entirely unexplored materials are proposed without a comprehensive characterization of their chemical and physical properties. Consequently, nanomaterials are under “special” observation by health regulatory agencies, which are taking appropriate measures to characterize their effects in a biological context fully. Such a monitoring pathway is even more necessary considering that materials, at the nanoscale, show entirely different properties with respect to their bulk counterparts. Thus, their toxicological profile needs more careful evaluations that often are not envisioned by standard evaluation protocols.

Challenges in nanomaterial biosafety evaluation include elaborating complex and dedicated *in vitro* models, high-throughput testing, and even predictive computational models. The more urgent questions that hinder widespread exploitation of innovative (piezoelectric) nanomaterials for biomedical applications are related to long-term adverse effects, their fate in the organisms and the environment, and a careful evaluation of the acceptability of their benefit/risk ratio. To answer these questions, a tight collaboration among researchers, regulatory authorities, and clinicians is mandatory. A possible intriguing route regards the adoption of highly biocompatible and biodegradable piezoelectric vectors. For this purpose, recent works demonstrate the potential of nanoparticles made of piezoelectric nylon-11 and nanotubes made of polylactic acid as relatively soft interfaces for mediating intracellular phenomena.^70,126^

It is clear that the biomedical research on piezoelectric nanomaterials has now reached a development step in which all of the mentioned safety assessment phases have to be considered to translate their applications toward realistic exploitations. The findings collected in the research of recent years are promising, but it is time to bring piezoelectric nanomaterials to the next step of bio- and immunocompatibility testing.

### Control of the US Dose Released *in Vivo*

As mentioned in the previous sections, the authors consider it crucial to find, *in vitro*, the optimal US dose able to trigger the desired bioeffects. However, this is useless if then such an optimal dose cannot be translated *in vivo* in the desired target area within the human body. This is absolutely not straightforward and currently represents an open challenge in this field.

Indeed, the acoustic waves must be correctly tuned to cross heterogeneous tissues, while delivering the desired activating US acoustic dose in the targeted area, especially if it is located deep in the body (Figure 5g). Reflections, attenuations, diffractions, and other physical phenomena can affect and distort the US beam, especially if some tissues (*e.g.*, bones, lungs, and fat) characterized by acoustic properties rather different from the other soft tissues are present along the acoustic path. Therefore, the “*in vitro* to *in vivo* translation” of the US dose triggering specific bioeffects is challenging but crucial to reproduce *in vivo* the beneficial effects found *in vitro*.

Analytical models and FEM analyses could help in predicting the patient-specific acoustic propagation of the US. This knowledge may provide information about the correct positioning of the transducer used for stimulation and a correct retuning of its output power for reaching the target with the desired US dose.

Analytical solutions for wave propagation have been proposed for different US sources. Most of them start from the Rayleigh–Sommerfeld integral, which gives the solution for a plane, axisymmetric vibrating surface.^127^ However, they are limited by simplifications such as low intensities, simple geometries, and linear approximation. More complex analyses, also taking into account nonlinear effects, can be addressed only by numerical methods.^128^ The k-wave Matlab toolbox^129^ is probably the most widely used US simulation software nowadays. It is based on a numerical model that solves the main partial differential acoustic equations in the k-space frequency domain.^130^ FEM methods through dedicated (*e.g.*, PZFlex Virtual Prototyping) or multiphysics (*e.g.*, Comsol) software, have also been recently proposed thanks to the advancements of computing capability.^127^

Beyond modeling efforts, the integration of real-time image guidance strategies is fundamental for an effective, safe, and precise *in vivo* delivery of the US energy. This especially applies to therapies in which the spatial localization of the US-induced effects is an essential requirement. Current treatment guidance solutions rely on MRI, which provides high-resolution images and the possibility to control temperature elevation. Another technology for treatment guidance is US echography used at a high acquisition frame rate (around 30–40 Hz in standard commercial systems).

Another important aspect, which is usually overlooked during *in vivo* US applications, is the verification of an adequate acoustic coupling between the transducer and the human/animal body. Such a coupling is commonly performed by putting the transducer in contact with the patient skin through water-based gel coupling media. If this procedure is not performed correctly, the energy transmission within the body and, consequently, the activating US dose in the target changes dramatically. Therefore, to minimize undesired reflections, more attention should be paid to this aspect and, whenever possible, online monitoring solutions should be implemented.^131^

Furthermore, real-time monitoring *in situ* of the US-induced physical effects (*e.g.*, thermal enhancement measurements,^132^ acoustic cavitation monitoring,^133^*etc.*) during the *in vivo* procedure should also be performed whenever possible to directly confirm and control the efficacy and safety of the therapy, avoiding a “blind” stimulation.

## Possible Future Routes and Conclusion

Today, a few research reports are available on the use of piezoelectric nanomaterials and US to modulate drug delivery: this application domain is still relatively unexplored. However, it may have an enormous impact on the biomedical community. In 2015, Vannozzi *et al.* developed an ultrathin polymeric film composed of polylactic acid and polyelectrolytes (chitosan and hyaluronic acid) with embedded BTNPs. They investigated how a US stimulus could modulate the release of an antirestenosis drug embedded within the polyelectrolytes layers.^110^ A boost of drug release upon US stimulation (40 kHz US source) and a significant difference in the drug release kinetics with respect to the control groups (nonpiezoelectric and non-US stimulated films) was found. Such an effect was mainly due to piezoelectric and mechanical effects, whereas thermal ones did not contribute. The presence of piezoelectric nanoparticles probably led to the generation of localized charges upon US application, thus triggering the opening of the polyelectrolyte network thanks to the motion of ions and a consequent increment of the released drug. In 2018, Timin and colleagues explored the use of a piezoelectric polymer (poly(3-hydroxybutyrate)), loaded with silica microcapsules embedding bioactive molecules (*e.g.*, bovine serum albumin) onto the scaffold surface.^134^ Here, the presence of piezoelectric properties in the matrix resulted in an alteration of the surface charges, favoring the adhesion of a higher number of bioactive molecules. A comparative analysis among triggering by US, enzymes, and laser radiation was performed, demonstrating a faster response in terms of modulation of the drug kinetics when subjected to US (20 kHz, 50 W for 120 s).

Although the examples mentioned above demonstrate the high potential of using drug-loaded piezoelectric nanocomposite platforms and US stimulation for a modulable and triggerable release of drugs, this target application is scarcely explored to date. This route would deserve greater attention in the future. For example, nanocomposite piezoelectric patches may be sutured after a surgical operation (*e.g.*, postmyocardial infarction or post-tumor resection), injected in cavities (such as the knee, for cartilage regeneration purposes), or delivered by endoscopic capsules in endoluminal cavities (*e.g.*, stomach, intestine, to treat bowel diseases or to act on the equilibrium of the intestinal microbiota). Then, they may be triggered by US to locally release chemotherapeutics or other drugs in a controlled way. This would allow activating the desired effect with a temporized release profile, selected on-demand by the patient or the clinician.

Furthermore, strategies for more precise targeting of piezoelectric nanomaterials toward specific cell types *in vivo* may have a tremendous impact, especially concerning the treatment of cancer cells and neurodegenerative diseases. As a relevant example, the targeting of D1 neurons of the basal ganglia with piezoelectric nanoparticles and their subsequent US-assisted stimulation would allow the accurate activation of the direct pathway in patients with Parkinson’s disease and the consequent alleviation of their motor symptoms. A similar approach has been previously demonstrated in mice using optogenetic tools.^135^ Other relevant nonmotor symptoms in Parkinson’s disease, such as pain hypersensitivity,^136^ might also be attenuated through the piezostimulation of basal ganglia circuitry.

In conclusion, the combination of piezoelectric nanoparticles and US stimulation showed up in the last two decades as a possible game-changing approach in several biomedical fields, such as neuromodulation, regenerative medicine, cancer therapy, and beyond. Despite significant amount of evidence accumulated so far especially *in vitro*, in-depth knowledge of the physical and biological mechanisms lying behind them is still missing. As a consequence, the optimization of this approach and its clinical translation must pass through a series of steps, addressing (*i*) piezoelectric properties measurement, (*ii*) control of the US dose delivered *in vitro*; (*iii*) quantification of US-mediated piezo effects; (*iv*) deep understanding of the biological mechanisms behind the triggered bioeffects; (*v*) therapy targeting strategies; (*vi*) biocompatibility studies; and (*vii*) control of the US dose delivered *in vivo*. Some of these pieces of the puzzle are at a low level of maturation; others have already reached a relatively high technological readiness level, although intended for different scientific or clinical purposes. Hopefully, future research efforts will allow for significant steps ahead in all of the above-mentioned domains, thus transforming this scientifically exciting paradigm into a clinically viable technology.

## Vocabulary

**controlled ultrasound**: well-characterized ultrasound field which enables the precise correlation between the applied dose and the biological findings
**LIPUS**: therapeutic ultrasound regime characterized by a noncontinuous wave and relatively low pressures which do not induce lethal effects on cells
**piezoelectric nanomaterial**: material with nanometric dimensions able to become electrically polarized when they are mechanically stimulated and *vice versa*
**neuromodulation**: modulation of neural activity in the central or peripheral nervous system
**regenerative medicine**: branch of medicine that aims to engineer or regenerate cells, tissues, or organs
